# Postoperative cerebral infarction after revascularization in patients with moyamoya disease: Incidence and risk factors

**DOI:** 10.3389/fneur.2022.1053193

**Published:** 2022-11-21

**Authors:** Jiaxiong Wang, Hanqiang Jiang, Jinwei Tang, Chi Lin, Wei Ni, Yuxiang Gu

**Affiliations:** ^1^Department of Neurosurgery, South Yunnan Central Hospital of Yunnan Province (The First People's Hospital of Honghe Prefecture), Mengzi, China; ^2^Department of Neurosurgery, Huashan Hospital, Shanghai Medical College, Fudan University, Shanghai, China

**Keywords:** moyamoya disease, surgical revascularization, complication, cerebral infarction, risk factor

## Abstract

**Objectives:**

Cerebral infarction is the major complication of revascularization surgery in patients with moyamoya disease (MMD), and we analyzed the possible causes of cerebral infarction after revascularization surgery for MMD.

**Methods:**

MMD patients who were admitted and underwent surgical revascularization at Shanghai Huashan Hospital from January 2019 to December 2021 were retrospectively analyzed.

**Results:**

A total of 815 patients and 890 revascularization surgeries (677 first revascularization surgeries and 213 second revascularization surgeries) were included in this study; 453 (50.9%) were performed on the left side and 437 (49.1%) on the right side, with 779 (87.5%) combined procedures and 111 (12.5%) indirect bypasses included. The mean patient age at the time of these procedures was 44.6 ± 11.7 years (range 6–72 years). Postoperative cerebral infarctions were observed in 46 (5.17%) surgeries, among which 31 occurred after left hemisphere revascularization surgeries, with an incidence of 6.84%, and 15 occurred after right hemisphere revascularization surgeries, with an incidence of 3.43%. Of these, 30 (65.2%) occurred in the operated hemispheres, 2 (4.3%) in the contralateral hemisphere and 13 (28.3%) in the bilateral hemisphere. There were 11 cases of massive infarction (23.9%). The incidence of postoperative infarction in patients undergoing the first revascularization was 6% (41/677) and 2.3% (5/213) in the second revascularization surgeries. Initial presentation as infarction (*P* < 0.001), initial presentation as hemorrhage (*P* < 0.001), hypertension (*P* = 0.018), diabetes (*P* = 0.006), 1st or 2nd surgery and surgical side (*P* = 0.007) were found to be related to postoperative cerebral infarction. Initial presentation as infarction (OR = 2.934, 95% CI 1.453–5.928, *P* = 0.003), initial presentation as hemorrhage (OR = 0.149, 95% CI 0.035–0.641, *P* = 0.011), and 1st or 2nd surgery and surgical side (OR = 1.66, 95% CI 1.106–2.491, *P* = 0.014) were independently associated with cerebral infarction after revascularization surgeries.

**Conclusions:**

In patients with MMD undergoing surgical revascularization, initial presentation as infarction and first revascularization surgery performed on the left hemisphere are independent risk factors for postoperative cerebral infarction, whereas initial presentation as hemorrhage is a protective factor.

## Introduction

MMD is a bilateral steno-occlusive disorder of the intracranial terminal internal carotid artery (ICA) and proximal middle cerebral artery (MCA)/anterior cerebral artery (ACA) vessels, with the development of abnormal vascular networks. In clinical manifestations, MMD involves ischemic and hemorrhagic processes through a variety of pathophysiological mechanisms. Presently, no medical treatment has been shown to be effective in halting the natural progression of MMD. Interventional endovascular stenting has failed to prevent future ischemic events and does not halt MMD progression (1). The most important way to treat MMD is surgical revascularization, but the surgical procedure itself is dangerous. Adverse postoperative events occurred in 9.3–12.9% of cases, 62.5–71.4% of which were cerebral infarctions (2–6). Postoperative cerebral infarction is one of the most severe complications, resulting in disability and dysfunction. The cause of postoperative cerebral infarction, however, remains obscure. The objective of this study was to analyze the predictors of postoperative acute-phase (within 7 days) infarction in the revascularization of MMD. In the present study, the medical records of Huashan Hospital were reviewed to determine the causes of postoperative cerebral infarction.

## Materials and methods

### Patient selection

This retrospective study was approved by the institutional Ethics Committee of Huashan Hospital, Fudan University. The electronic hospital records of patients with MMD admitted to and undergoing surgical revascularization at Huashan Hospital from January 2019 to December 2021 were reviewed retrospectively. Patients were eligible for further analyses if (1) they were diagnosed with MMD (2) they underwent surgical revascularization, and (3) they had complete medical records. The diagnosis criteria of MMD was according to 2021 revised version issued by Research Committee on Moyamoya Disease (Spontaneous Occlusion of the circle of Willis) (7). Cerebral angiography revealed stenosis or occlusion at the terminal portion of intracranial internal carotid artery with moyamoya vessels at the base of brain. Unilateral cases with risk factors of atherosclerotic disease were excluded from the study. Moreover, patients with coagulation disorders, hyperthyroidism, systemic lupus erythematosus, antiphospholipid antibody syndrome, nodular peripheral arteritis, and Sjogren's syndrome were also excluded from the study.

### Preoperative evaluation and management

Basic characteristics, including age, sex, presenting symptoms, surgical modalities, and radiological patterns, were collected. A history of vascular risk factors, including hypertension and diabetes mellitus, was also recorded. DSA or MRA/CTA imaging was used to determine the Suzuki stage. Antiplatelet therapy was discontinued 7 days before surgery for patients with ischemic MMD. Patients with recent symptoms of TIA were bridged with low molecular weight heparin (LMWH), and LMWH was discontinued 1 day before surgery. In patients with hemorrhagic MMD, a dose of hemostatic agents is administered immediately after the surgery. Postoperatively, all patients received intravenous infusion therapy. Anticoagulation and antiplatelet therapy were generally not used during the acute postoperative phase.

### Surgical strategy and procedure

Patients who developed stroke (ischemic or haemorrhagic) or TIA were treated with revascularization of the hemisphere responsible for the manifestations. For non-stroke patients who presented with cognitive impairment, headache, dizziness, etc., surgery was performed on the more severely hypoperfused hemisphere, which was determined by arterial spin labeling (ASL) imaging or CT perfusion. The criteria of hypoperfusion status revealed by ASL or CT perfusion was described as follows: (1) ASL: regional cerebral blood flow (rCBF) decrease with arterial transit artifacts (ATA). (2) CT perfusion: rCBF decrease with prolonged regional mean time to transit (rMTT) and time to peak (rTTP). In the case of symmetrical hypoperfusion patients, revascularization was performed on the dominant hemisphere side. Contralateral surgery was recommended 6 months later for all patients. Surgical procedures, including direct, indirect and combined bypass, were described in our previous study (8, 9). In brief, the direct bypass procedure was an end-to-side anastomosis of the superficial temporal artery (STA) to identical cortical branches of the middle cerebral artery (MCA). An indirect bypass was performed using encephalo-dura-myo-synangiosis (EDMS). Combined revascularization is defined as simultaneous anastomosis of STA-MCA and EDMS. The skin flap incision was curved posteriorly to preserve as much of the integrity of the posterior branch of the STA as possible. After anastomosis, patency was confirmed by Doppler ultrasound and indocyanine green (ICG) angiography. Combined bypass was always the preferred option for the patients (8). If there was no suitable donor or recipient vessel, EDMS was performed directly.

### Perioperative management

During surgery, blood pressure was maintained over baseline value to prevent cerebral hypoperfusion. After surgery, blood pressure was strictly controlled to avoid cerebral hyperperfusion syndrome. Intraoperative bleeding was controlled <100 ml. Blood transfusion was determined by the concentration of blood haemoglobin and disease severity. Enough sedation was given based on the fluid balance, cardiac function and body weight. An individualized dehydration treatment was devised depending on symptoms and brain edema revealed by CT or MRI scan. We use mannitol and diuretics for postoperative neurological symptoms. Hyperventilation was avoided to prevent cerebral ischemia in patients with poor cerebrovascular reserve (CVR) as hyperventilation was a stimulus for vasodilation like acetazolamide.

### Postoperative cerebral infarction

Cerebral infarction that occurred within 1 week after surgery should be confirmed by postoperative imaging, such as CT or diffusion-weighted imaging (DWI).

### Statistical analysis

IBM SPSS Statistics 24.0 software was used to conduct all statistical analyses. Participants were divided into two groups based on the occurrence of cerebral infarction after revascularization. Categorical variables are presented as frequencies, and the differences were compared with the chi-square test or Fisher's exact test. Normally distributed continuous variables are presented as the mean ± SD, and comparisons were performed using a *t*-test. We calculated odds ratios and 95% confidence intervals for potential risk factors for postoperative infarction by univariate logistic regression and binomial regression analyses. The statistical tests were all two-sided, and *P* < 0.05 was considered to indicate statistical significance.

## Results

### Baseline characteristics

A total of 815 patients (397 males and 418 females) were enrolled in the study. Among them, 75 patients had undergone two revascularization operations for the bilateral hemispheres, 138 patients had undergone revascularization surgery on one hemisphere before this study, and a second revascularization surgery on the contralateral side was performed during this study period. Overall, 890 revascularization surgeries (677 first revascularization surgeries and 213 second revascularization surgeries) were included in this study; 453 (50.9%) were performed on the left side and 437 (49.1%) on the right side, with 779 (87.5%) combined procedures and 111 (12.5%) indirect bypasses included. The mean patient age at the time of these procedures was 44.6 ± 11.7 years (range 6–72 years). The baseline clinical characteristics are summarized in Table 1.

**Table 1:** Baseline characteristics of the included surgeries.

**Characteristic**	**All cases**	**Percentage (%)**
No. of surgeries	890	100
Mean age at the time of surgery	44.6 ± 11.7	
**Sex**		
Male	432	48.5
Female	458	51.5
**Initial presentation**		
Infarction	327	36.7
Hemorrhage	289	32.5
TIA	111	12.5
Headache, dizziness, and others	163	18.3
**Concomitant diseases**		
Hypertension	147	16.5
Diabetes	27	3
Hypertension and diabetes	55	6.2
**Surgery type**		
Combined	779	87.5
Indirect bypass	111	12.5
**Suzuki stages of surgical sides**		
3	115	12.9
4	349	39.2
5	303	34
6	123	13.8
**First revascularization surgeries**		
Left hemisphere	349	39.2
Right hemisphere	328	36.9
**Second revascularization surgeries**		
Left hemisphere	104	11.7
Right hemisphere	109	12.2

TIA, transient ischemic attack.

### New cerebral infarction after revascularization surgery

Graft patency of all the direct bypasses were confirmed by intraoperative indocyanine green angiography (ICGA). Radiologically confirmed postoperative cerebral infarctions were observed in 46 (5.17%) surgeries, among which 31 occurred after left hemisphere revascularization surgeries, with an incidence of 6.84%, and 15 occurred after right hemisphere revascularization surgeries, with an incidence of 3.43%. After further analysis, 30 (65.2%) occurred in the operated hemispheres, 2 (4.3%) occurred in the contralateral hemisphere, and 13 (28.3%) occurred in the bilateral hemisphere. Of the 46 infarctions, two (ID 8 and ID 44) occurred after two surgeries for the same patient. In total, 11 (23.9%) of the 46 infarctions were massive infarctions. The In 8 of 11 patients with massive infarctions, preoperative angiogram revealed that severe stenosis or even near total occlusion of the MCA at operated hemisphere with collateral compensation via retrograde blood flow from the impaired ACA. In patients with small infarction or without complications, most of the MCA feeding territories were supplied by anterograde flow from impaired MCAs. Forty-one cerebral infarction events occurred in patients undergoing the first revascularization, and the incidence was 6% (41/677). Overall, five infarctions occurred after the second revascularization surgery, and the incidence was 2.3% (5/213), all in the operated hemispheres. The angiographic grade [according to Matsushima neoangiogenesis grading system (10)] of the first revascularization of these five patients was grade A in four cases and grade B in 1 case. The details of patients with postoperative complications of infarction are presented in Table 2. In terms of clinical outcomes of the patients with postoperative cerebral infarctions, two deaths and nine moderate-severe disabilities were observed in 11 patients with massive infarctions. Moreover, neurological worsening was observed in 8 of 35 patients with small infarctions after surgery, and the incidence was 22.8%.

**Table 2:** Details of patients with postoperative infarction complications.

**ID**	**Sex**	**Age**	**Initial presentation**	**Disease duration (month)**	**Concomitant diseases**	**Suzuki** **stage**		**Surgical side**	**Post-operative** **infarction**	
						**R**	**L**		**Side**	**Size**
**1st revascularization**									
1	M	53	Dizziness	1		4	6	L	B	Small
2	M	47	Infarction	36	Hypertension	6	5	L	L	Small
3	M	43	Infarction	1		6	4	L	L	Small
4	M	46	Infarction	24		4	4	R	B	Small
5	F	44	Infarction	2	Hypertension	5	5	R	R	Small
6	M	40	Infarction	3	Hypertension, DM	5	0	R	R	Small
7	M	43	TIA	1		4	4	L	B	Small
8	M	45	Infarction	0.5		2	3	L	L	Small
9	F	44	Infarction	3	Hypertension, DM	6	5	L	L	Small
10	F	64	Infarction	12	Hypertension	4	5	R	B	Small
11	F	49	Infarction	3	Hypertension	5	5	L	L	Small
12	F	48	TIA	3	DM	5	4	L	L	Small
13	F	31	Infarction	2		5	3	L	B	Small
14	F	47	Infarction	1		5	4	L	R	Small
15	M	67	Infarction	120	Hypertension, DM	4	6	L	L	Small
16	F	63	Hemorrhag	4		0	4	L	L	Small
17	F	54	Infarction	2	DM	3	5	L	B	Small
18	M	46	Infarction	4	Hypertension	5	5	L	L	Small
19	F	44	Infarction	4		4	4	L	B	Small
20	M	53	Infarction	6	DM	2	5	L	L	Small
21	F	62	Infarction	1		5	4	R	R	Small
22	F	66	asymptomatic	12	Hypertension	4	4	L	L	Small
23	M	52	Infarction	3		1	5	L	B	Small
24	M	50	Infarction	12		4	4	R	B	Small
25	M	63	Infarction	5		4	4	L	L	Small
26	M	36	TIA	5	DM	6	5	L	L	Small
27	M	54	Hemorrhag	4		4	4	L	B	Small
28	M	32	Infarction	5		4	3	R	R	Small
29	M	47	Infarction	3	Hypertension	6	6	L	L	Small
30	M	59	Infarction	2		4	6	L	L	Small
31	F	53	Infarction	10		5	5	R	R	Small
32	F	49	Dizziness	1		5	5	L	L	Massive
33	F	54	Infarction	12		4	4	L	L	Massive
34	F	56	Infarction	2	DM	4	4	R	R	Massive
35	M	50	TIA	2	Hypertension	3	5	L	L	Massive
36	M	40	Infarction	1	Hypertension	4	4	L	B	Massive
37	M	35	TIA	3		4	4	R	B	Massive
38	M	49	Infarction	12	Hypertension	5	4	R	B	Massive
39	M	50	Infarction	24	DM	6	6	L	L	Massive
40	F	55	Headache	24		4	4	R	R	Massive
41	F	47	TIA	2		5	4	L	R	Massive
**2nd revascularization**									
42	F	38	Infarction	36		5	4	L	L	Small
43	M	36	Infarction	0.7		4	6	R	R	Small
44	M	45	Infarction	7		4	5	R	R	Small
45	M	39	Infarction	48	Hypertension	3	5	R	R	Small
46	M	40	Infarction	9	Hypertension,DM	4	4	L	L	Massive

F, female; M, male; L, left; R, right; B, bilateral; TIA, transient ischemic attack; DM, diabetes mellitus.

### Factors associated with postoperative cerebral infarction

Binomial logistic regression analysis was performed to explore the association between the presence of postoperative cerebral infarction and clinical variables. According to univariate analysis, initial presentation as infarction (*P* < 0.001), initial presentation as hemorrhage (*P* < 0.001), hypertension (*P* = 0.018), diabetes (*P* = 0.006), 1st or 2nd surgery and surgical side (*P* = 0.007) were found to be related to postoperative cerebral infarction. Although there were no statistically significant differences, we observed a higher incidence of postoperative cerebral infarction in male patients than in female patients, 6.25 and 4.1%, respectively (*p* = 0.157), and the incidence of postoperative infarction in patients aged <20 years, 20–40 years, 40–60 years, and over 60 years was 0, 3.58, 5.8, and 9.1%, respectively (*p* = 0.091). Additionally, combined bypass was more likely to cause infarction than indirect bypass alone, with incidence rates of 5.5 and 2.7%, respectively (*p* = 0.24) (Table 3).

**Table 3:** Demographic characteristics and risk factor analysis of postoperative infarction.

**Characteristics**	**Post-operative infarction**		***p*-value**
	**Yes** **(*n* = 844)**	**No** **(*n* = 46)**	
No. of surgeries	844	46	
**Age group, years**			0.074
≤ 20	30 (3.6%)	0 (0%)	
> 20 ≤ 40	269 (31.9%)	10 (21.7%)	
> 40 ≤ 60	485 (57.5%)	30 (65.2%)	
>60	60 (7.1%)	6 (13.1%)	
**Sex**			0.157
Male	405 (48%)	27 (58.7%)	
Female	439 (52%)	19 (41.3%)	
**Initial presentation**			< 0.001^*^
Infarction	293 (34.7%)	34 (73.9%)	< 0.001^*^
Hemorrhage	287 (34%)	2 (4.3%)	< 0.001^*^
TIA	105 (12.4%)	6 (13%)	0.904
Headache, dizziness, and others	159 (18.8)	4(8.7%)	0.083
**Concomitant disease**			
Hypertension	185 (21.9%)	17 (36.9%)	0.018^*^
Diabetes	72 (8.5%)	10 (21.7%)	0.006^*^
**Surgery type**			0.21
Combined	736 (87.2%)	43 (93.5%)	
Indirect bypass	108 (12.8%)	3 (6.5%)	
**Suzuki stages of surgical sides**			0.346
3	112 (13.3%)	3 (6.5%)	
4	326 (38.6%)	23 (50%)	
5	288 (34.1%)	15 (32.6%)	
6	118 (14%)	5 (10.9%)	
**1st or 2nd surgery and surgical side**			0.007^*^
1st and left	320 (37.9%)	29 (63%)	0.001^*^
1st and right	316 (37.4%)	12 (26.1%)	0.12
2nd and left	102 (12.1%)	2 (4.3%)	0.112
2nd and right	106 (12.6%)	3 (6.5%)	0.224

* Statistical significance (*p* < 0.05).

Second, we performed binary logistic regression including these variables, which were found to be related to postoperative cerebral infarction. The results showed that initial presentation as infarction (OR = 2.934, 95% CI 1.453–5.928, *P* = 0.003), initial presentation as hemorrhage (OR = 0.149, 95% CI 0.035–0.641, P = 0.011), 1st or 2nd surgery and surgical side (OR = 1.66, 95% CI 1.106–2.491, P =0.014) were independently associated with cerebral infarction after revascularization surgeries. Risk factors included the first revascularization surgery performed on the left hemisphere and initial presentation as infarction, but initial presentation as hemorrhage was protective (Table 4).

**Table 4:** Logistic regression analysis for predictive factors of postoperative infarction.

**Characteristics**	**OR**	**95% CI**	***p*-value**
**Initial presentation**			
Infarction	3.09	1.49–6.39	0.002^*^
Hemorrhage	0.16	0.038–0.70	0.015^*^
**Concomitant disease**			
Hypertension	1.46	0.71–2.97	0.3
Diabetes	1.77	0.76–4.11	0.19
First and Left revascularization surgery	1.65	1.09–2.49	0.019^*^

* Statistical significance (*p* < 0.05).

## Discussion

Surgical revascularization is the most effective treatment option for MMD. Both symptomatic and asymptomatic MMD patients may benefit from revascularization surgery (1, 11). Postoperative cerebral infarction is still a challenging issue for MMD patients after revascularization, which impedes clinical outcomes due to neurological deterioration and long-term deficits. This study reviewed 890 revascularization procedures for MMD, and postoperative cerebral infarction occurred in 46 cases, with an incidence of 5.17%, which is in agreement with the rates reported in previous literature (1, 12–14).

**Figure 1:**
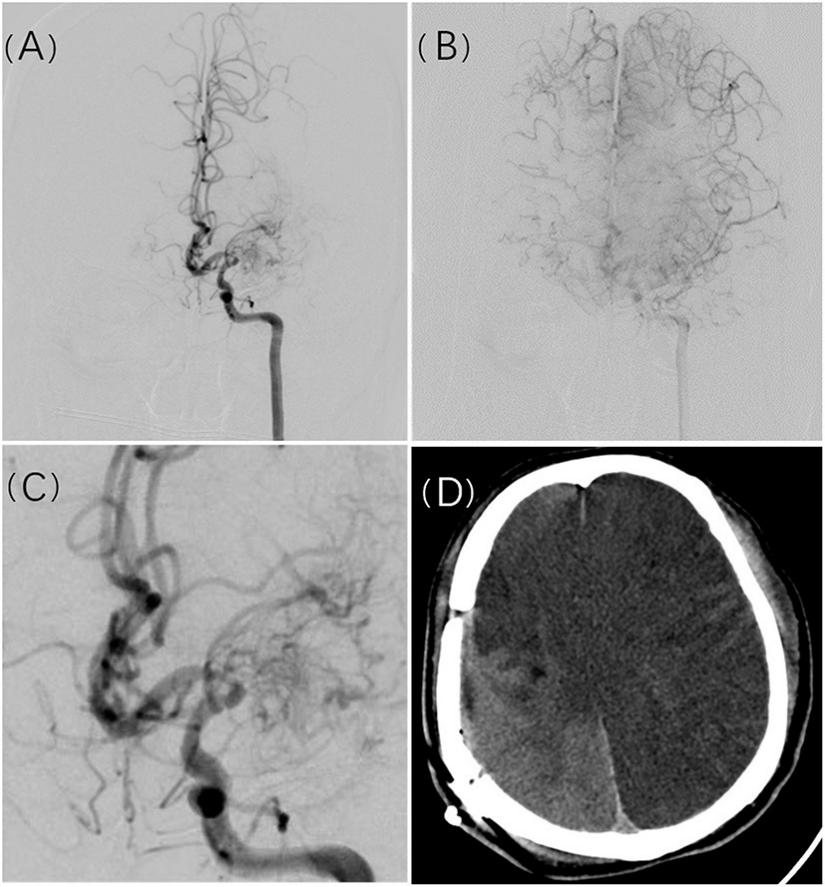
A 49-year-old man presented with recurrent slurred speech and numbness of the left limbs. Cerebral angiogram revealed left MCA occlusion with compensation to the right ACA territory **(A,B)**. Manification of the L ICA angiogram showed severe stenosis of A1 **(C)**. Postoperative massive cerebral infarction occurred in the bilateral hemisphere approximately 3 days after surgical revascularization **(D)**.

Our results suggest that initial presentation as infarction and the first revascularization surgery performed on the left hemisphere are independent risk factors for postoperative cerebral infarction. Initial presentation as hemorrhage is a protective factor. In theory, vessel diameter correlates positively with the size of its feeding area. Either cerebrovascular stenosis (absolute stenosis), blood-stealing phenomenon (increased blood flow burden), or both occurring together may lead to insufficient cerebral blood flow reserve, increasing the likelihood of systemic hemodynamic changes causing cerebral infarction. In 8 of 11 patients with massive cerebral infarction, DSA showed extreme stenosis or even near total occlusion of the MCA, while its feeding area was compensated via retrograde flow from the impaired ACA. In patients with complications of small volume infarction or without complications, most of the MCA feeding territories were supplied by anterograde flow from impaired MCAs. We believe that patients with massive infarction had a manifestation of collateral compensatory blood supply deficiency and regional hemodynamic instability (Figure 1). Furthermore, no correlation was found between the location of new infarcts and the surgical site. Of 41 cases with infarction occurred after the first revascularization, 16 were on the contralateral or bilateral side, and the remaining 25 occurred on the surgical side, but 11 of these were distant from the surgical site. Hara et al. (15) discussed the imaging pattern and the mechanisms of postoperative infarction after indirect revascularization in patients with MMD. This suggested that postoperative infarction that occurred in the cortex under the craniotomy site might be caused by direct irritation of the cortex during the surgical procedure, and widespread cortical infarction that extends outside the craniotomy site seems to be caused by postoperative hemodynamic fluctuation. In addition, it has been proven that high variance in intraoperative blood pressure and drastic blood pressure decline are independent risk factors for postoperative infarction in MMD patients who underwent revascularization surgery (16). From the literature and our observations, systemic pathophysiological changes caused by surgery and anesthesia may induce cerebral infarction rather than the procedure itself.

Postoperative cerebral infarction was an in-reversible complication which differed from focal cerebral hyperperfusion or watershed shift ischemia. In this study, all the patients would receive CT or MRI scan if symptoms occurred after revascularization surgery. If cerebral infarction was detected, cerebral perfusion examination was not routinely arranged, as some patients could not cooperate well, especially those with massive infarction. For patients without positive findings on CT or MRI scan, ASL examination would be arranged to identify the etiology. As described in literature, symptomatic cerebral hyperperfusion dominated over 20% in patients receiving direct bypass surgery (17). A significant increase of blood flow around the site of anastomosis was usually observed by perfusion imaging. However, the symptoms could relieve in a majority of patients after strict blood control, dehydration and administer of oxygen free radical scavenger. Recently, watershed shift ischemia phenomenon was another mechanism which might be responsible for the symptoms after direct bypass surgery (18). With the dynamic change of CBF after bypass, sometimes the retrograde flow from the donor could decrease the anterograde flow and induced hypoperfusion in the remote territory of the MCA (18). Regrettably, a lack of perfusion imaging for most patients with postoperative cerebral infarction existed in this study. We speculated that the hemodynamic change after bypass possibly brougt improper watershed shift ischemia. Cerebral infarction might occur if the reverse flow of bypass dominated over anterograde flow from the severe stenosis vessels, such as ACA.

MMD manifests as either ischemic or hemorrhagic based on variations in the characteristic pattern of the vascular network at the base of the brain. The vast majority of patients with cerebral infarction and cerebral hemorrhage began their illness without a clear cause, leading us to believe that the patient's systemic hemodynamics did not fluctuate during the onset. The cause of cerebral infarction is insufficient spontaneous collateral compensation, whereas intracerebral hemorrhage indicates abundant collateral compensation. The pathogenesis of hemorrhagic MMD is presumed to be the rupture of collateral vessels or the formation of an aneurysm as a result of an excessive hemodynamic load. In other words, initial presentation as infarction and initial presentation as hemorrhage may reflect the deficiency or adequacy of the cerebrovascular network, which facilitates the redistribution of cerebral blood flow during hemodynamic changes. Patients with initial presentation as hemorrhage are therefore more tolerant of hemodynamic changes caused by anesthesia and surgery. In a previous study (13), it was determined that ischemic presentation prior to revascularization is an independent risk factor for subsequent ischemia. Patients who present with preoperative ischemia are more likely to have inadequate collateralization pathways to compensate for hemodynamic impairment. Surgical procedures may induce stress on the regulation of hemodynamics, resulting in ischemic complications. Normal cerebral perfusion was found to be associated with hemorrhage in other studies of hemorrhagic MMD (19); thalamic anastomosis, choroidal anastomosis, and periventricular anastomosis play a crucial role in the hemorrhagic presentation of MMD (20–22). Revascularization may improve the dilation and branch extension of AChA-PCoA and reduce the rate of re-bleeding by decreasing the collateral circulation load (9).

MMD is typically a bilateral hemispheric disease requiring staged revascularization surgery on both hemispheres. Generally, the side with symptoms or the side with hypoperfusion should be operated on first. Most surgeons will first consider operating on the dominant hemisphere of asymptomatic patients discovered by chance. Interhemispheric differences in postoperative infarction, as well as differences between the first and second revascularization, are not well-documented. Our research revealed differences between these two points. Due to the interaction of the left and right sides with the first and second surgeries, we compared four conditions: first surgery on the left side, first surgery on the right side, second surgery on the left side, and second surgery on the right side. The results indicate that initial left-sided revascularization is an independent risk factor for postoperative infarction.

Consistent with the findings of a previous study, our findings indicate that revascularization procedures conducted on the left hemisphere are more likely to result in postoperative cerebral infarction (23). Surgery conducted in the dominant hemisphere is more likely to cause systemic symptoms such as aphasia, cognitive loss, and affective disorders, as well as heart rate and blood pressure variations. The left hemisphere is typically the dominant hemisphere. Hypovolemia due to the use of mannitol and diuretics for postoperative neurological symptoms is also more common after the operation on the left side than on the right. In addition, the right common carotid artery originates from the brachiocephalic trunk (typically at a right angle to the flow of the innominate artery), whereas the left artery originates directly from the aortic arch and runs more in a straight line with the ascending aorta. This anatomical difference contributes to the difference in hemodynamics between the left and right carotid arteries. The systemic hemodynamic changes have a more direct effect on the left internal carotid artery's hemodynamics. In contrast, the innominate artery acts as a buffer for the right carotid artery, regulating blood flow dynamics. The hemodynamic impacts associated with the specific anatomy of the carotid vessels may explain the preferential occurrence of cerebral infarction on the left side (24, 25).

**Figure 2:**
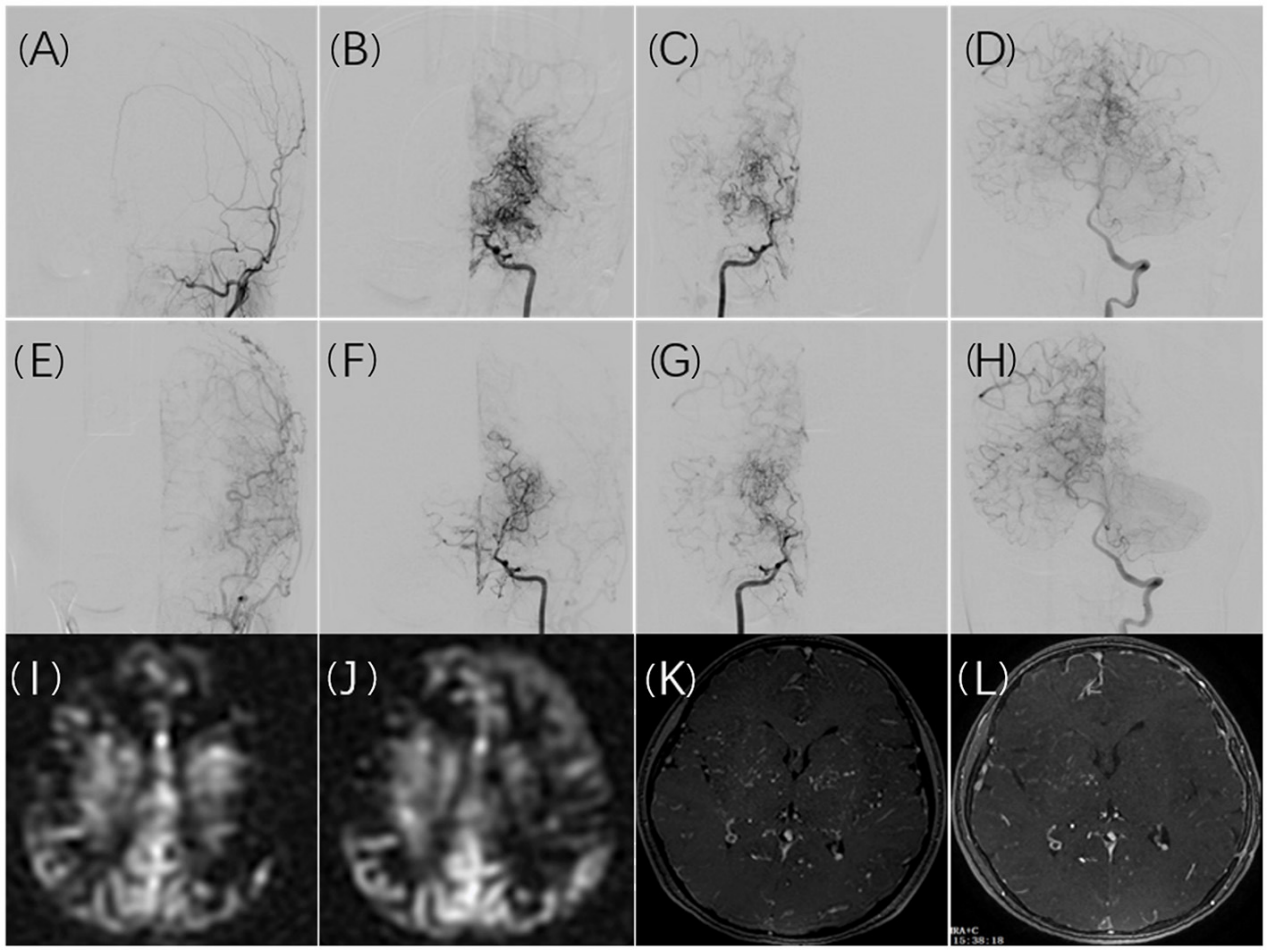
A 10-year-old girl presented with recurrent numbness of the right limbs. Cerebral angiography revealed bilateral moyamoya disease **(B,C)** without collateral anastomosis for the left ECA **(A)**. Collateral compensation from the posterior circulation was noted **(D)**. Six months after surgical revascularization, the left ECA angiogram demonstrated graft patency and good angiogenesis **(E)**. Shrinkage of moyamoya vessels on both hemispheres was noted **(F,G)**, and the left internal carotid artery compensated for the right hemisphere **(F)**. The compensatory range of the posterior circulation shifted to the non-operated territory **(H)**. The MRI ASL images showed hypoperfusion in the cerebral cortex **(I)**. The hypoperfusion in the left cortex improved after surgical revascularization with a decrease in perfusion in the left basal ganglia **(J)**. Ivy collateral vessels in contrast MRA decreased obviously in the left and right basal ganglia after surgical revascularization **(K,L)**.

A significant difference was observed between the incidence of cerebral infarction following the first revascularization and the incidence following the second revascularization. This may be partly because the first surgery is more likely to be performed in the symptomatic hemisphere. However, some findings in the literature as well as our study led us to conclude that the first revascularization procedure improves cerebral blood flow reserve, which allows patients to better tolerate hemodynamic instability in the second operation. After the first surgery, 16 (39%) of the 41 infarct events occurred at contralateral hemispheres. However, there were no infarct events in the contralateral hemisphere after the second surgery. According to imaging examinations performed before the second revascularization surgery, cerebral blood flow and cerebral perfusion had improved to varying degrees in most postoperative hemispheres, and bilateral improvement had been noted in some patients. As reported in a study published in 2021 (26), cerebral reactivity and TIA frequency in the contralateral hemisphere can be improved following unilateral revascularization surgery in bilateral MMD. Figure 2 illustrates the imaging changes observed in patients with bilateral MMD after unilateral surgery in a typical case. Revascularization significantly improved blood supply to the postoperative hemisphere, resulting in a redistribution of blood flow, the cerebral vascular supply range shifting from the postoperative hemisphere to the un-operated hemisphere, and a uniform cerebral perfusion pattern. The dilated and disordered vascular morphology of the bilateral cerebral hemispheres was also partially restored.

Our study has some limitations. First, our study was conducted retrospectively at a single center, and the recruitment of cases was not consecutive, which led to bias in patient selection. Second, our study only evaluated the incidence of cerebral infarction during the postoperative period of the patients, but we did not assess additional complications, such as bleeding or epileptic seizures, which may have been associated with the occurrence of infarction. Third, as immediate DSA or further hemodynamic imaging was not performed on patients who experienced postoperative infarction, the mechanisms underlying the subsequent stroke episode were uncertain.

## Data availability statement

The raw data supporting the conclusions of this article will be made available by the authors, without undue reservation.

## Ethics statement

The studies involving human participants were reviewed and approved by Institutional Ethics Committee of Huashan Hospital, Fudan University. The patients/participants provided their written informed consent to participate in this study.

## Author contributions

JW: concepts and design and manuscript writing. HJ: data acquisition, and analysis. JT and CL: manuscript review. WN: manuscript preparation and editing. YG: supervision. All authors contributed to the article and approved the submitted version.

## Funding

This study was supported by Grant No. 2019YSY076 from Shanghai Municipal Health Commission.

## Conflict of interest

The authors declare that the research was conducted in the absence of any commercial or financial relationships that could be construed as a potential conflict of interest.

## Publisher's note

All claims expressed in this article are solely those of the authors and do not necessarily represent those of their affiliated organizations, or those of the publisher, the editors and the reviewers. Any product that may be evaluated in this article, or claim that may be made by its manufacturer, is not guaranteed or endorsed by the publisher.
